# Supplementary material for: Alteration of Autophagy and Glial Activity in Nilotinib-Treated Huntington’s Disease Patients
Source: Metabolites. 2022 Dec 6;12(12):1225. doi: 10.3390/metabo12121225 (PMC9781133; doi:10.3390/metabo12121225)
Supplement: Supplementary file 1 [file metabolites-12-01225-s001.zip › metabolites-2050817-supplementary.pdf]

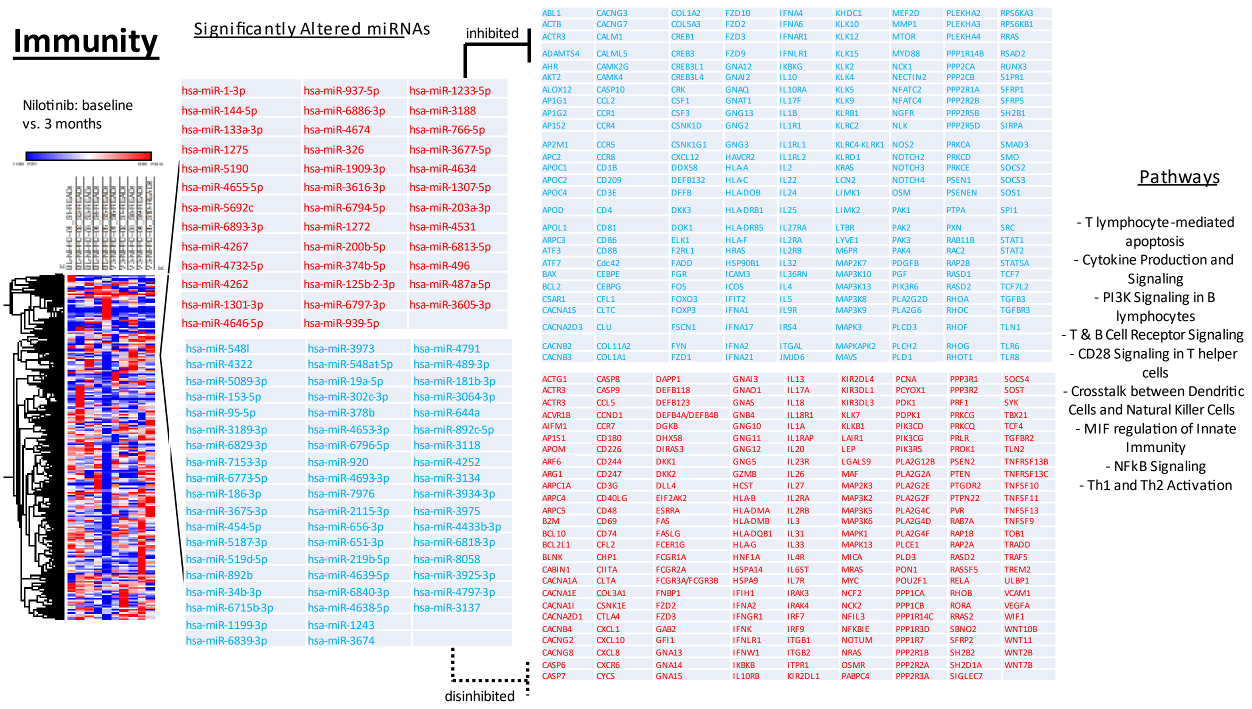

Figure S1. miRNAs targeting genes associated with innate and adaptive immunity are altered following nilotinib treatment. Gene ontology analysis revealed 38 upregulated and and 55 downregulated miRNAs that target 476 genes experimentally observed to be associated with immunological processes, including B and T cell receptor signaling, inflammatory cytokine production, and innate immune cell crosstalk.

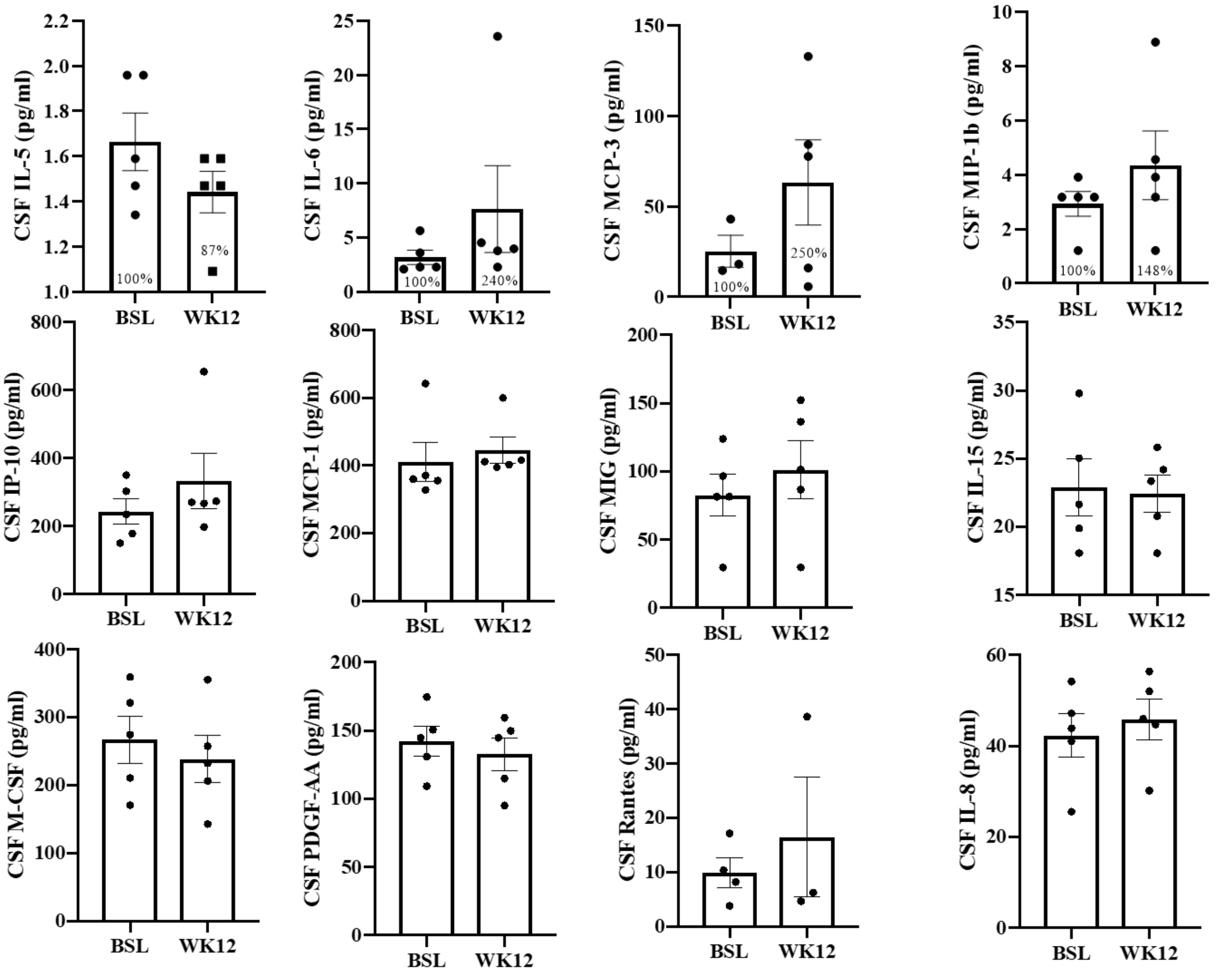

Figure S2. ELISA concentrations inflammatory markers in the CSF following nilotinib treatment.

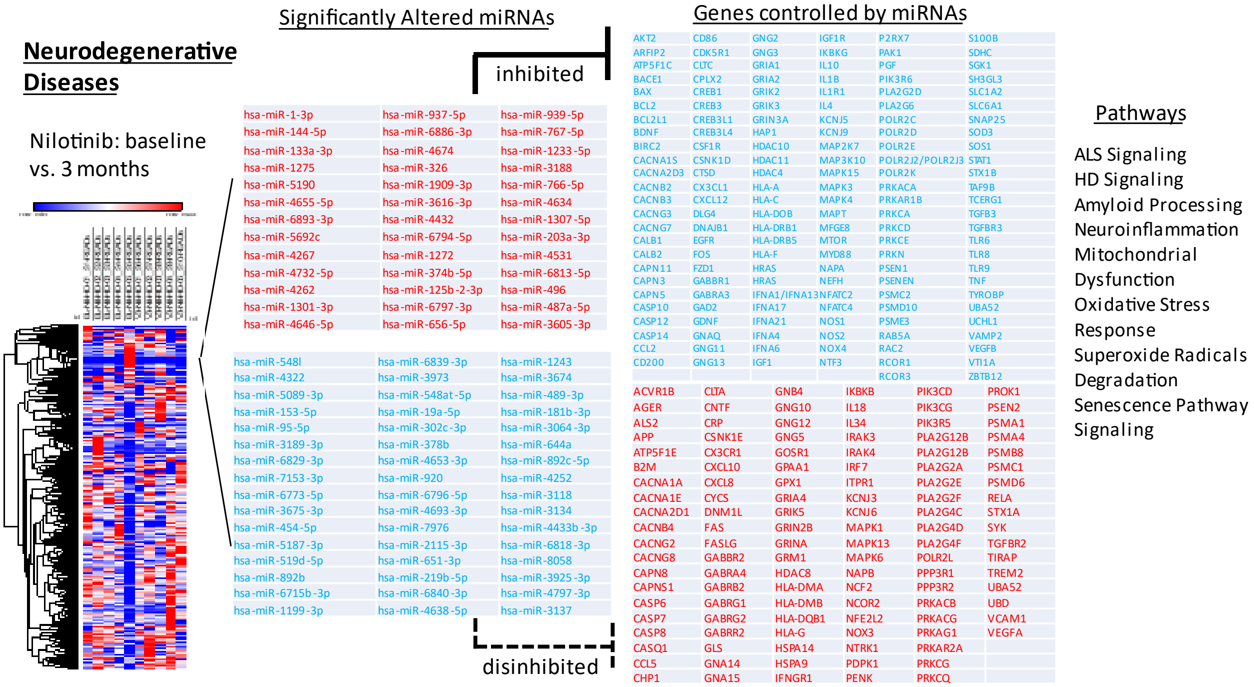

Figure S3. miRNAs targeting genes associated with various neurodegenerative disorders are altered following nilotinib treatment. Gene ontology analysis revealed 39 upregulated and 48 downregulated miRNAs that target 268 genes experimentally observed to be associated with neurodegenerative diseases including AD, PD, ALS, and HD.

Table S1. Demographics and Adverse Events.

Patient ID	Nil-HD-001	Nil-HD-002	Nil-HD-003	Nil-HD-004	Nil-HD-005	Nil-HD-006	Mean +/- SD
**Age**	62	47	61	63	58	50	56.83 ± 6.74
**Gender**	M	F	M	F	M	M	
**Weight (kg)**	84.1	58.7	65.3	82.9	82.13	97.1	78.37 ± 13.97
**Height (cm)**	177.3	171.8	178	158.9	178.2	176	172.58 ± 8.07
**BMI**	26.8	19.89	20.61	32.83	25.86	31.35	26.11 ± 5.95
**Race**	White	White	White	White	White	White	
**Ethnicity**	Not Hispanic or Latino	Hispanic or Latino	Not Hispanic or Latino	Not Hispanic or Latino	Not Hispanic or Latino	Not Hispanic or Latino	
**Selective serotonin reuptake inhibitor**	Zoloft (Sertraline)	-	-	-	-	Prozac (Fluoxetine)	
	**No. AEs (%)**	**No. AEs (%)**	**No. AEs (%)**	**No. AEs (%)**	**No. AEs (%)**	**No. AEs (%)**	**Total No. of Adverse Events (%)**
**Psychiatric Disorders**	Irritability 1 (5.2%)					Obsessive compulsive disorder 1 (5.2%)	2 (10.5%)
**Nervous System Disorders**	Headache 1 (5.2%)	Headache 1 (5.2%)		Headache 1 (5.2%)			3 (15.8%)
**Skin Disorders**	Folliculitis 2 (10.5%)						2 (10.5%)
**Digestive System Disorders**		Stomach pain 1 (5.2%)					1 (2.5%)
**Respiratory Tract Disorders**		Sinus 1 (5.2%)<br>Runny nose (5.2%)	Flu 1 (5.2%)	Flu 1 (5.2%)			4 (21%)
**General Disorders**			Fatigue 1 (5.2%)	Cold sore 1 (5.2%)<br>Dry skin 2 (10.5%)<br>Fatigue 1 (5.2%)<br>Allergies 1 (5.2%)		Motion sickness 1 (5.2%)	7 (36.84%)
**No. of AEs per patient (%)**	4 (21%)	4 (21%)	2 (10.5%)	7 (36.84%)	0 (0%)	2 (10.5%)	19 (100%)

EKG QTc interval (ms)

Patient ID	Screening	Baseline	0.5 Month	1 Month	1.5 Month	2 Month	3 Month	4 Month
Nil-HD-001	440	426	438	417	435	427	424	415
Nil-HD-002	459	433	426	432	447	438	442	Remote visit
Nil-HD-003	389	400	400	410	Remote visit	Remote visit	416	403
Nil-HD-004	414	425	413	Remote visit	Remote visit	Remote visit	431	428
Nil-HD-005	422	423	425	417	426	416	418	422
Nil-HD-006	403	402	407	384	408	410	408	388

Table S2. Exploratory Clinical Outcomes.

	Baseline (Mean ± SD)	3 Mths (Mean ± SD)	4 Mths (Mean ± SD)	BSL Vs 3 Mths (p value)	3 Mths Vs 4 Mths (p value)
**MOCA**	26.50 ± 2.74	25.83 ± 3.0	28.4 ± 1.14	0.25	0.1563
**Timed up and go**	10.05 ± 2.0	12.02 ± 2.38	10.25 ± 1.64	0.1563	0.0781
**TMT-B**	120.2 ± 79.15	120 ± 74.69	116.2 ± 72.44	0.5	0.5
**UHDRS-m (motor score)**	28 ± 13.89	27.33 ± 13.63	28.33 ± 10.88	0.375	0.125
**Apathy (Partner)**	21.5 ± 4.37	23 ± 5.1	21.83 ± 4.79	0.125	0.4531
**Apathy (Participant)**	23 ± 3.69	26.17 ± 2.32	24.17 ± 2.4	0.1875	0.125
**Irritability (Partner)**	14.17 ± 4.79	15.83 ± 4.22	17.17 ± 4.12	0.3125	0.3281
**Irritability (Participant)**	15.17 ± 8.04	17 ± 9.14	19 ± 6.03	0.375	0.4063
